# Extracellular matrix derived from Wharton’s Jelly-derived mesenchymal stem cells promotes angiogenesis via integrin αVβ3/c-Myc/P300/VEGF

**DOI:** 10.1186/s13287-022-03009-5

**Published:** 2022-07-18

**Authors:** Beilei Ma, Tengkai Wang, Juan Li, Qian Wang

**Affiliations:** 1 Department of Clinical Laboratory, Qilu Hospital, Cheeloo College of Medicine, Shandong University, Jinan, 250012 Shandong China; 2 Department of Gastroenterology, Qilu Hospital of Shandong University, Jinan, 250012 Shandong China; 3 Department of Clinical Laboratory, Qilu Hospital of Shandong University (Qingdao), Qingdao, 266035 China

**Keywords:** Angiogenesis, Mesenchymal stem cells-derived extracellular matrix, Wharton's Jelly-derived mesenchymal stem cells, Vascular endothelial growth factor, Histone acetylation level

## Abstract

**Background:**

Angiogenesis is required in many physiological conditions, including bone regeneration, wound healing, and tissue regeneration. Mesenchymal stem cells-derived extracellular matrix (MSCs-ECM) could guide intricate cellular and tissue processes such as homeostasis, healing and regeneration.

**Methods:**

The purpose of this study is to explore the effect and mechanism of ECM derived from decellularized Wharton's Jelly-derived mesenchymal stem cells (WJ-MSCs) on endothelial cell viability and angiogenesis. The human umbilical vein endothelial cells (HUVECs) were pretreated with WJ-MSCs ECM for 2d/7d/14d, respectively. After pretreatment, the angiogenesis ability of HUVECs was detected.

**Results:**

In this study, we found for the first time that WJ-MSCs ECM could improve the angiogenesis ability of HUVECs with a time-dependent manner in vitro. Mechanically, WJ-MSCs ECM activated the focal adhesion kinase (FAK)/P38 signaling pathway via integrin αVβ3, which further promoted the expression of the cellular (c)-Myc. Further, c-Myc increased histone acetylation levels of the vascular endothelial growth factor (VEGF) promoter by recruiting P300, which ultimately promoting VEGF expression.

**Conclusions:**

ECM derived from Wharton’s Jelly-derived mesenchymal stem cells promotes angiogenesis via integrin αVβ3/c-Myc/P300/VEGF**.** This study is expected to provide a new approach to promote angiogenesis in bone and tissue regeneration.

## Introduction

Angiogenesis begins with the dissolution of the basement membrane, followed by the migration and attachment of endothelial cells toward the extracellular space, and consequently forms an endothelial sprout [1, 2]. It is required in many physiological conditions, including bone regeneration, wound healing, and tissue regeneration [3]. In the case of bone regeneration, osteogenesis is coupled with angiogenesis during bone formation [4]. Angiogenesis facilitates local blood flow to bone tissue. The adequate blood supply is essential for bone regeneration to transport nutrients, oxygen, minerals, and metabolic waste [5]. Accumulated evidences suggest that in the absence of a functional and adequate vasculature network, bone tissue will undergo necrosis and bone formation failure [6]. Therefore, promoting angiogenesis during tissue repair will facilitate the regeneration of bone and tissue.

Mesenchymal stem cells (MSCs) have drawn lots of attention as gold standard stem cells in fundamental and clinical researches during the last 20 years. MSCs have been found to favor angiogenesis through supporting EC function via paracrine mechanism [7]. MSCs-derived extracellular matrix (MSCs-ECM) is comprised of a complex and highly organized macromolecular network of biomolecules mostly derived from stem cells [8]. Studies have confirmed that MSCs-ECM could guide intricate cellular and tissue processes such as homeostasis, healing and regeneration [9]. Wharton's Jelly-derived mesenchymal stem cells (WJ-MSCs) are acquired from umbilical cord tissue with the ability to secrete and synthesize a large amount of endogenous ECMs [10–12]. The ECM derived from WJ-MSCs contains abundant collagenic structural proteins, and other ECM components such as fibronectin and laminin [13]. It has been shown to have positive effects on cell viability and function. The ECM derived from WJ-MSCs could promote hepatic differentiation of human induced pluripotent stem cell [14]. Besides, it also improves the behavior of cells from degenerated intervertebral disc [15]. However, there is still no study about the effect of ECM derived from decellularized WJ-MSCs on endothelial cell viability and angiogenesis.

Integrin is the main receptor through which ECM regulates cell function. There are 24 integrin heterodimers which are generated from 18 α integrin subunits and 8 β integrin subunits combination [16]. Among them, integrin αVβ3, integrin α5β1 and integrin α5β5 are the main receptors for ECM regulation of endothelial cell function [17]. In this study, we harvested ECM from passage 4 WJ-MSCs, and treated human umbilical vein endothelial cells (HUVECs) with the ECM. We aimed to explore the effect and mechanism for the ECM derived form WJ-MSCs on endothelial cell angiogenesis by detecting the changes in HUVECs viability and function as well as the expression of integrin-related genes. This study is expected to provide a new approach to promote angiogenesis in bone and tissue regeneration.

## Materials and methods

### Chemicals and reagents

The antibodies of vascular endothelial growth factor (VEGF) (A8074), focal adhesion kinase (FAK) (A11131), phospho-FAK-Y397 (p-FAK) (AP0302), P38 (A14401), phospho-P38 (AP0055), Collagen I (COL I) (A16891) and cellular (c)-Myc (A1309) were obtained by Abclonal Inc. (China). The antibody of P300 (sc-48343) was obtained by Santa Cruz Inc. (USA). SiRNA for c-Myc and P300 were purchased from Sigma (USA). FAK inhibitors (PF-573228), antagonist for integrin αVβ3 (HY-100563) and integrin α5β1 (HY-13535A) were obtained by MCE Inc. (USA). DMEM/F12 (1:1), PBS and fetal bovine serum were provided by Gibco (St. Louis, Missouri, USA).

### Isolation and culture of human WJ-MSCs

Human WJ-MSCs were isolated as previously described [18]. Briefly, MSCs were isolated from collected human umbilical cords within 2 h. Removing the umbilical arteries and umbilical vein, Wharton’s jelly was peeled off from the remaining part of the umbilical cords and transferred to a sterile container and then cut into pieces smaller than 0.5 cm^3^. The minced Wharton’s jelly was digested for 4 h in a 50-ml sterile centrifuge tube with 30-ml culture medium containing collagenase of type I (Invitrogen, Thermo Fisher Scientific Inc., USA) at 0.2% in an incubator (5% carbon dioxide, 37 °C). After centrifuging the liquid at 300 × g for 15 min and discarding the supernatants, the cells were resuspended in DMEM/F12 medium with 10% fetal bovine serum and 1% penicillin–streptomycin (Gibco BRL, Thermo Fisher Scientific Inc., USA) in humidified air with 5% carbon dioxide at 37 °C. The WJ-MSCs were passaged once the flask reached approximately 80% confluence and the fourth passage was used for the next experiments.

### Preparation of ECM

The ECM derived from WJ-MSCs was prepared as previously described [19]. Briefly, plastic flasks (Plastic) were precoated with 0.2% gelatin (Sigma-Aldrich, St. Louis, MO) at 37 °C for 1 h and seeded with passage 4 WJ-MSCs at 6,000 cells per cm^2^. After reaching 90% confluence, cells were cultured for another 10 days with 250 μM L-ascorbic acid phosphate (Wako Chemicals USA, Inc., Richmond, VA). The cells were lysed with 0.5% Triton X-100 containing 20 mM ammonium hydroxide at 37 °C for 5 min and then stored at 4 °C in PBS containing 100 U/mL penicillin, 100 μg/mL streptomycin, and 0.25 μg/mL fungizone until use.

### Culture and treatment for HUVECs

HUVECs were purchased from American type cultures (Rockville, MD, USA) and cultured in endothelial cell growth medium (ECM, ScienCell, USA) containing 5% FBS and supplemented with endothelial cell growth supplement (ECGS, ScienCell, USA). HUVECs at passage 3 to 5 were used in this experiment. For the pretreatment of HUVECs with WJ-MSCs ECM, the culture plates were precoated with WJ-MSCs ECM before seeding HUVECs. For the control group, the HUVECs were cultured in normal plates without ECM for 14d. For the ECM group of 2d, the HUVECs were cultured in normal plates without ECM for 12d, and then the HUVECs were transferred into plates precoated with WJ-MSCs ECM for 2d. For the ECM group of 7d, the HUVECs were cultured in normal plates without ECM for 7d, and then the HUVECs were transferred into plates precoated with WJ-MSCs ECM for 7d. For the ECM group of 14d, the HUVECs were cultured in plates precoated with WJ-MSCs ECM for 14d. All the groups were cultured in plates for 14d. As to the expression silence of genes, HUVECs were transfected with SiRNA, and Lipofectamine 3000 (Invitrogen, Carlsbad, CA, USA) was used at 50 nM in the final episode.

### Assays for detecting the angiogenesis ability of HUVECs

To detecting the angiogenesis ability of HUVECs, HUVECs were pretreated with ECM for 2d/7d/14d, respectively. After pretreatment, 1.0 × 10^5^ HUVECs were harvested and seeded in the upper chamber of the 12-well transwell culture plate (8 μm, Corning, NY, U.S.A.) with serum-free medium, and the lower chamber contains a culture medium containing serum. After 24 h culture, the upper cells were removed and stained with 20% methanol and 0.2% crystal violet. The cells are then imaged under an optical microscope (Olympus), with 10 separate field counts inserted. The results were the average of the three independent experiments. For the tube formation, the treated HUVECs (1 × 10^4^/well) were seeded into Matrigel-coated wells in a 96-well plate. The cells were seeded in endothelial cell growth medium (ECM, ScienCell, USA) without serum for 8 h. Only a tube that was completely continuous between two branching points was considered a tube.

### RNA isolation and RT-qPCR

Total RNA was isolated using TRIzol® reagent. Simply placed HUVECs in a 1.5 mL homogenizer, and added 1 ml TRIzol to fully homogenate, and let stand at room temperature for 5 min. After adding 0.2 ml chloroform, the homogenizer was centrifuged for 15 s, and left standing for 2 min. After centrifugation at 4 °C for 12,000 g × 15 min, the supernatant was placed in another 1.5 mL centrifuge tube. 0.5 ml isopropyl alcohol was added to the centrifuge tube, and the centrifuge tube was left standing at room temperature for 10 min after gently stirring. After centrifugation at 4 °C for 12,000*g*×10 min, the supernatant was discarded. Using 1ML 75% ethanol, wash the precipitate gently. After centrifugation at 4 °C for 7500*g*×5 min, the supernatant was discarded. After drying, DEPC H_2_O was added to dissolve (65 °C, ~ 10–15 min). 1 μg RNA was transcribed into cDNA. RT-qPCR was performed on an A.B.I. Step One RT-qPCR hot circulator (A.B.I. Step One, New York, USA) using the SYBR®Premix Ex Taq™ kit. The RT-qPCR products isolated from the DNA extraction kit were diluted in multiples to construct the relative standard curve of the target gene. The results were expressed as fold values relative to the control group. The rat primer sequences of the genes used in this study are shown in Table 1.

**Table 1:** Oligonucleotide primers and PCR conditions in RT-qPCR

Genes	Forward primer (5′–3′)	Reverse primer (5′–3′)
VEGF	GCTCTACCTCCACCATGCCA	AGCTCATCTCTCCTATGTGC
Integrin αV	TGCATCAACCTTAGCTTCTGCCT	ACCAGCAGGCGGCTCTGGTTCAC
Integrin α5	GACAGGGAAGAGCGGGCACTATGG	GTCCCTTCCCGGCCGGTAAAACTC
Integrin β1	TGCCAGCCAAGTGACATAGAGA	ATCCGTTCCAAGACTTTTCACAT
Integrin β3	CTGCCGTGACGAGATTGAGT	TGCCCCGGTACGTGATATTG
Integrin β5	ACCTGGAACAACGGTGGAGA	AAAAGATGCCGTGTCCCCAA
β-Actin	CTACAATGAGCTGCGTGTGGC	CAGGTCCAGACGCAGGATGGC

*VEGF* vascular endothelial growth factor

### Chromatin immunoprecipitation (ChIP) assay

Cells in Alginate beads were cross-linked with 1% formaldehyde before sonicating in SDS lysis buffer. DNA in cell lysates was sheared to length of approximately 200 base pairs. Fragmented chromatin was first pre-cleared with protein A-sepharose 4B and rabbit IgG for 2 h. Before immunoprecipitating with fresh protein A-sepharose 4B and antibody include anti-histone 3 lysine 9 acetylation (H3K9ac), anti-H3K14ac and anti-H3K27ac (Abcam, USA) at 4 °C overnight. Sepharose beads were washed before eluting with 1% SDS followed by reverse cross-linking at 65 °C overnight. The samples were then placed in a 65 °C water bath overnight to reverse formaldehyde cross-linking and subsequently were purified using PCR purification kits. The isolated DNA was then assayed using RT-qPCR. The primer sequences of the promoters were as follows: forward, TACTTCCCCAAATCACTGTGG, reverse, CTCTGGAGCTCTTGCTACCTC. The input values were compared to the immunoprecipitated samples, with the IgG negative controls values subtracted as background. The calculated errors in all of the graphs depicting ChIP data represent the standard deviations for three replicate RT-qPCRs for precipitated chromatin, input chromatin, and background (i.e., chromatin precipitated with nonspecific IgG).

### Co-immunoprecipitation (Co-IP) assay

A Co-IP assay was performed to detect the interaction of c-Myc with P300. After washing with ice-cold PBS, cells were lysed in 1.2 ml lysis buffer at − 80 °C for 30 min. The samples were centrifugated with 14,000 rpm at 4 °C for 15 min and the supernatant was transferred to a new column. And then, the samples were divided into three parts: the first was used for input protein, the other two were performed using 1 ug of either a mock antibody (IgG) as control or c-Myc antibody, respectively, followed by incubation overnight at 4 °C with gentle shaking. And then the samples were incubated with protein G magnetic beads (Millipore, 16–157) at 4 °C for 6 h. After immunoprecipitation, the samples were washed with lysis buffer. After three washing pieces, retained proteins were mixed with 30 ul loading buffer at 100 °C for 10 min. Protein complexes and input protein were then detected by Western blotting.

### Immunofluorescence staining

For the immunofluorescence, cells were washed with PBS and fixed with 4% paraformaldehyde for 15 min. Cells were washed and permeated with 0.5% Triton X-100 in PBS for 20 min at room temperature, and then, the cells were blocked with 5% BSA in PBS for 1 h. Antibodies was added and incubated overnight at 4 °C. Fluorescent secondary antibody (1:100, Bioss Inc., Beijing, China) was added for 2 h at room temperature. Cells were then incubated with DAPI for 5 min at room temperature. Five sections of each slice were taken and photographed. The mean optical density (MOD) of immunohistochemistry was quantified by two individuals under the double-blind principle.

### Western blotting

About 0.04 mg protein was separated by 10% SDS-PAGE gel and adsorbed into the nitrocellulose membrane. The first-antibodies were incubated at 4 °C overnight. After incubating with the first-antibodies, the membrane was washed with TBST three times. Then, the membrane was incubated with a horseradish peroxidase-labeled secondary antibody (1:5000) at room temperature for 1 h. The signal was detected with ECL reagent.

### Statistical analysis

In this study, Prism 8.0 was used for data analysis. Quantitative data were expressed as mean ± S.E.M. Multiple comparisons were performed using one-way analysis of variance (ANOVA). The unpaired two-tailed student’s *t*-test was used for comparison between the control group and the other single groups. *P* < 0.05 was considered statistically significant.

## Results

### Identification and the composition of ECM derived from WJ-MSCs

**Fig. 1:**
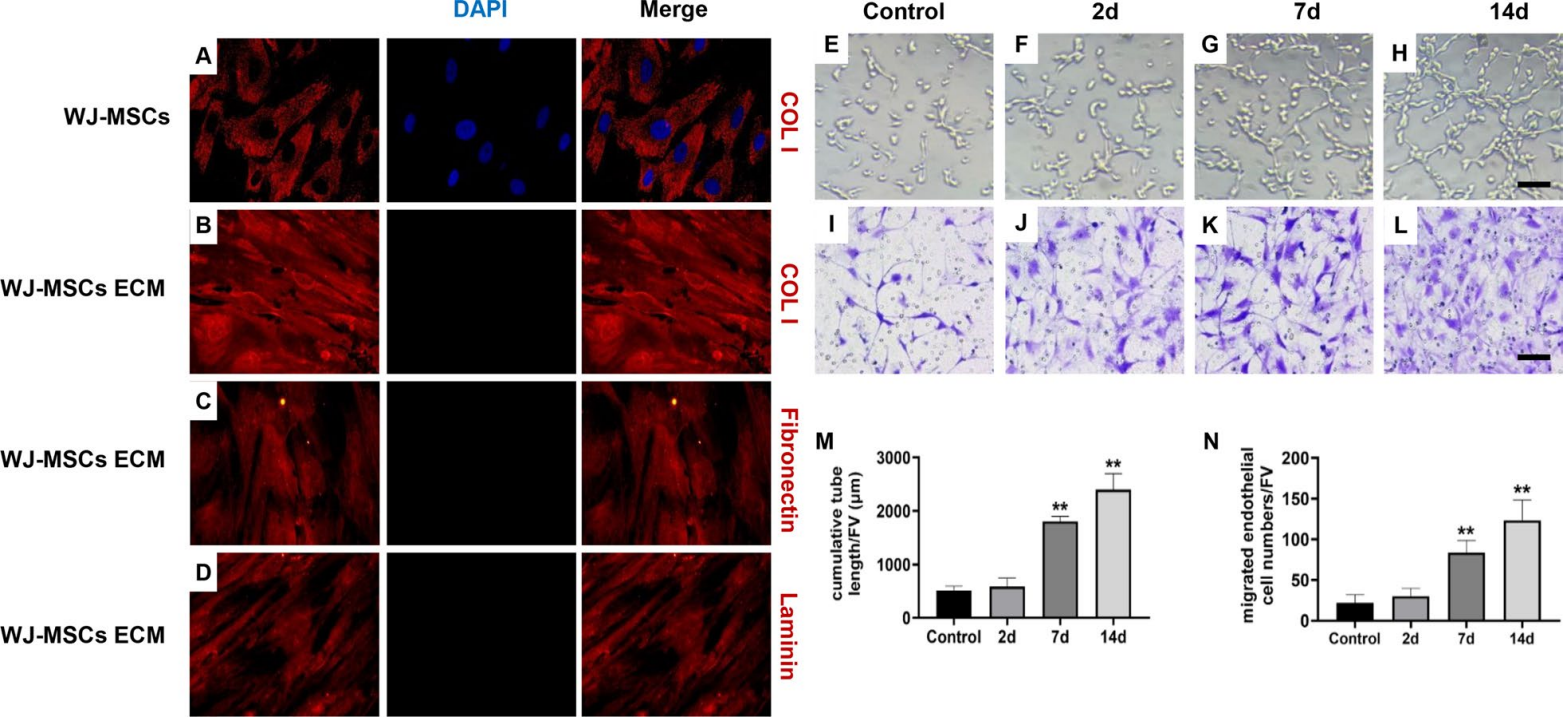
The effect of WJ-MSCs ECM on human umbilical vein endothelial cells (HUVECs) angiogenesis ability. **a, b** Immunofluorescently stained for collagen I (Col I) of WJ-MSCs or WJ-MSCs ECM (magnification: × 400).* n* = 3. **c** Immunofluorescently stained for Fibronectin of WJ-MSCs ECM (magnification: × 400).* n* = 3. **d** Immunofluorescently stained for Laminin of WJ-MSCs ECM (magnification: × 400).* n* = 3. **e**–**h**, **m** Matrigel tube formation assay images and quantitative analysis of cumulative tube length. Bar = 50 μm. Mean ± S.E.M., *n* = 3. **i**–**l**, **n**: Transwell for HUVECs migration assay and quantitative analysis of migrated HUVECs numbers. Bar = 50 μm. Mean ± S.E.M., *n* = 3. The *P* value was calculated using one-way ANOVA and independent samples *t*-test. *P < 0.05, **P < 0.01 versus control

To obtain ECM derived from WJ-MSCs, we conducted decellularization on cultured WJ-MSCs and identified the composition. It was found that the intact nuclear structure of WJ-MSCs disappeared by DAPI staining after decellularization (Fig. 1a, b). Further, by COL I staining which is the main component of WJ-MSCs cell matrix, we found that the intact cell membrane structure of WJ-MSCs disappeared after decellularization (Fig. 1a, b). The COL I escaped from the cell and dispersed in the outer stroma (Fig. 1b). Additionally, we identified the composition of ECM derived from WJ-MSCs. The results showed that ECM derived from WJ-MSCs expressed a large number of Fibronectin and Laminin (Fig. 1c, d). It was suggested that ECM derived from WJ-MSCs was successfully obtained by decellularization.

### The effect of WJ-MSCs ECM on HUVECs angiogenesis ability

To investigate the effect of WJ-MSCs ECM on endothelial cell, we inoculated HUVECs on normal culture plates (control group) or culture plates precoated with WJ-MSCs ECM (ECM group). For the control group, the HUVECs were cultured in normal plates without ECM for 14d. For the ECM group, the HUVECs were pretreated with WJ-MSCs ECM for 2d, 7d and 14d, respectively. The angiogenesis ability of HUVECs for the control group was measured by tube formation assay and migration assay after cultured for 14d. The angiogenesis ability of HUVECs for the ECM group was measured after being pretreated for 2d, 7d and 14d, respectively. The results showed that compared with normal culture plates, WJ-MSCs ECM significantly increased the cumulative tube length after being pretreated for 14d (Fig. 1e, h, m, *P* < 0.01) and the migrated number of HUVECs (Fig. 1i, l, n, *P* < 0.01). And that WJ-MSCs ECM increased the angiogenesis ability of HUVECs in a time-dependent manner (Fig. 1f–h, i–l, m–n, *P* < 0.01). These results suggested that WJ-MSCs ECM promoted HUVECs angiogenesis ability in a time-dependent manner.

### WJ-MSCs ECM increased HUVECs angiogenesis ability through integrin αVβ3/c-Myc/P300/VEGF signal

**Fig. 2:**
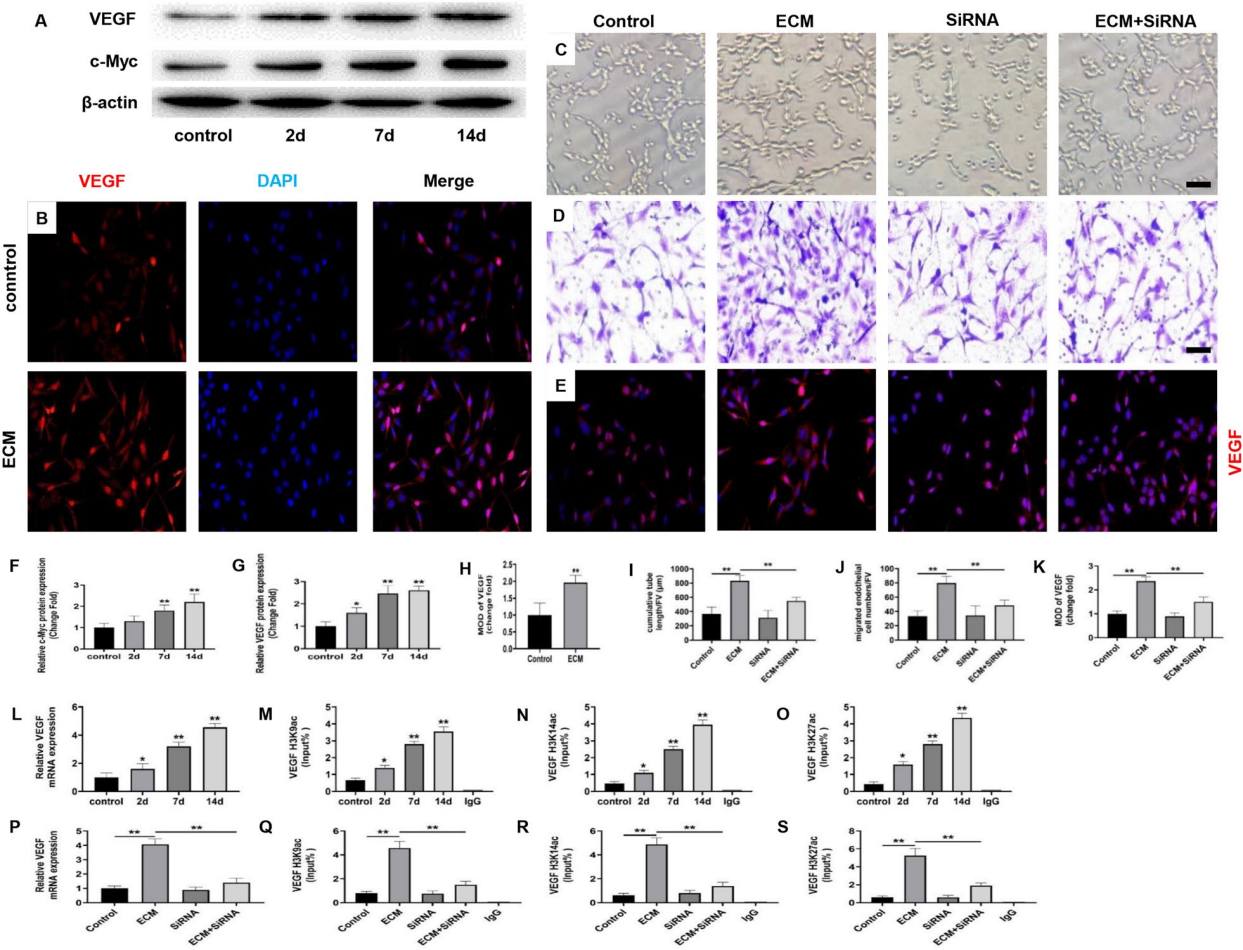
Cellular (c)-Myc mediated the increased expression of VEGF via histone acetylation modification in HUVECs induced by WJ-MSCs ECM. **a, f, g** Protein expression of VEGF and c-Myc in HUVECs was determined by western blotting. Mean ± S.E.M. *n* = 3. **b** Immunofluorescently stained for vascular endothelial growth factor (VEGF) of HUVECs (magnification: × 200). **h** The mean optical density (MOD) of VEGF expression was analyzed. Mean ± S.E.M. *n* = 3. **c, i** Matrigel tube formation assay images of cumulative tube length. Bar = 50 μm. Mean ± S.E.M., *n* = 3. **d, j** Transwell for HUVECs migration assay and quantitative analysis of migrated HUVEC numbers. Bar = 50 μm. Mean ± S.E.M., *n* = 3. **e, k** Immunofluorescently stained for VEGF of HUVECs (magnification: × 200). Bar = 50 μm. Mean ± S.E.M., *n* = 3. **l, p** mRNA expression of VEGF was determined by RT-qPCR. Mean ± S.E.M.,* n* = 5. **m**–**o**, **q**–**s** The H3K9ac, H3K14ac and H3K27ac levels in the VEGF promoter region were determined by RT-qPCR. Mean ± S.E.M., *n* = 5. SiRNA: c-Myc SiRNA. The *P* value was calculated using one-way ANOVA and independent samples *t*-test. *P < 0.05, **P < 0.01 versus control

Within the regulating factors, VEGF is one of the key regulators responsible for the angiogenic process. VEGF triggers the formation of neovascularization by stimulating endothelial cell migration [20, 21]. Therefore, to explore the mechanism for WJ-MSCs ECM promoted HUVECs angiogenesis ability, we detected the effect of WJ-MSCs ECM on the expression of VEGF by Immunofluorescent staining and Western Blotting. The results showed that WJ-MSCs ECM significantly promoted the protein expression VEGF (Fig. 2a, b, g, h, *P* < 0.05 or *P* < 0.01). The transcription factor c-Myc regulates the expression of several key genes related to angiogenesis [22–24]. Next, to find out whether c-Myc was involved in the increased expression of VEGF induced by WJ-MSCs ECM, we examined the effects of WJ-MSCs ECM on the expression of c-Myc. The results showed that ECM could promote the expression of c-Myc in HUVECs (Fig. 2a, f, *P* < 0.01). Furthermore, we silenced the expression of c-Myc in HUVECs by SiRNA and co-cultured it with WJ-MSCs ECM. The results showed that c-Myc expression silencing reversed the WJ-MSCs ECM-induced increase in HUVECs angiogenesis ability (Fig. 2c, d, i, j, *P* < 0.01) and VEGF expression (Fig. 2e, k, *P* < 0.01). These results suggested that c-Myc mediated the increased expression of VEGF induced by WJ-MSCs ECM.

Epigenetic regulation is a common gene regulation mode, which is crucial to maintaining organ function and normal life activities [25]. Among them, the histone acetylation is the most common epigenetic modification [26]. To explore the mechanism for c-Myc regulating VEGF expression, we detected the histone acetylation level of VEGF promoter region. And we found that the increased VEGF mRNA expression was accompanied by increased histone acetylation levels in the promoter region (Fig. 2l–o, *P* < 0.05 or *P* < 0.01). Furthermore, c-Myc expression silencing reversed the WJ-MSCs ECM-induced increase of VEGF mRNA and histone acetylation level in promoter region (Fig. 2p–s, *P* < 0.01). These results suggested that c-Myc might regulate the expression of VEGF through histone acetylation modification.

**Fig. 3:**
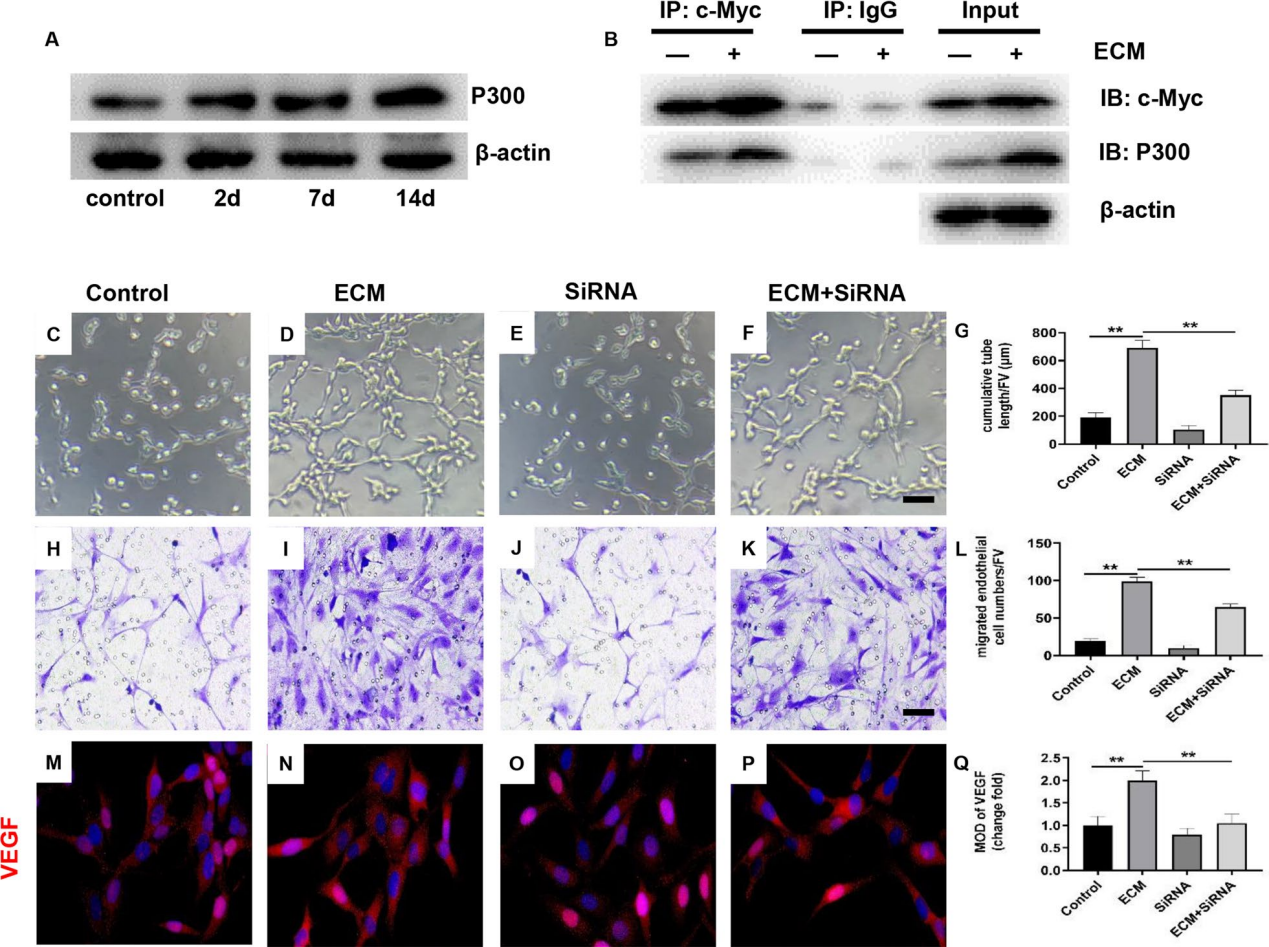
The interaction between P300 and c-Myc mediated the increased expression of VEGF in HUVECs induced by WJ-MSCs ECM. **a** Protein expression of P300 in HUVECs was determined by western blotting. Mean ± S.E.M. *n* = 3. **b** Analysis of the interaction between c-Myc and P300 by co-Immunoprecipitation (Co-IP). **c**–**g** Matrigel tube formation assay images of cumulative tube length. Bar = 50 μm. Mean ± S.E.M., *n* = 3. **h**–**l** Transwell for HUVECs migration assay and quantitative analysis of migrated HUVEC numbers. Bar = 50 μm. Mean ± S.E.M., *n* = 3. **m**–**q** Immunofluorescently stained for VEGF of HUVECs (magnification: × 400). Bar = 50 μm. Mean ± S.E.M., *n* = 3. SiRNA: P300 SiRNA. The *P* value was calculated using one-way ANOVA and independent samples *t*-test. *P < 0.05, **P < 0.01 versus control

To elucidate the mechanism regarding how c-Myc promotes histone acetylation levels in the promoter region of VEGF, we further examined the expression of histone acetylase P300 in HUVECs. The results showed that WJ-MSCs ECM promoted P300 expression in a time-dependent manner (Fig. 3a). Moreover, P300 expression silencing reversed the WJ-MSCs ECM-induced promotion of angiogenesis (Fig. 3c–l, *P* < 0.01), and increased expression of VEGF in HUVECs (Fig. 5m–q, *P* < 0.01). These results suggested that P300 was involved in the increase of VEGF expression induced by WJ-MSCs ECM. Further, we found that c-Myc interacted with P300 by Co-IP assay (Fig. 3b). Therefore, it’s suggested c-Myc promoted VEGF expression of HUVECs by recruiting P300 and increased the histone acetylation level of VEGF promoter region in the occasion of treating with WJ-MSCs ECM.

**Fig. 4:**
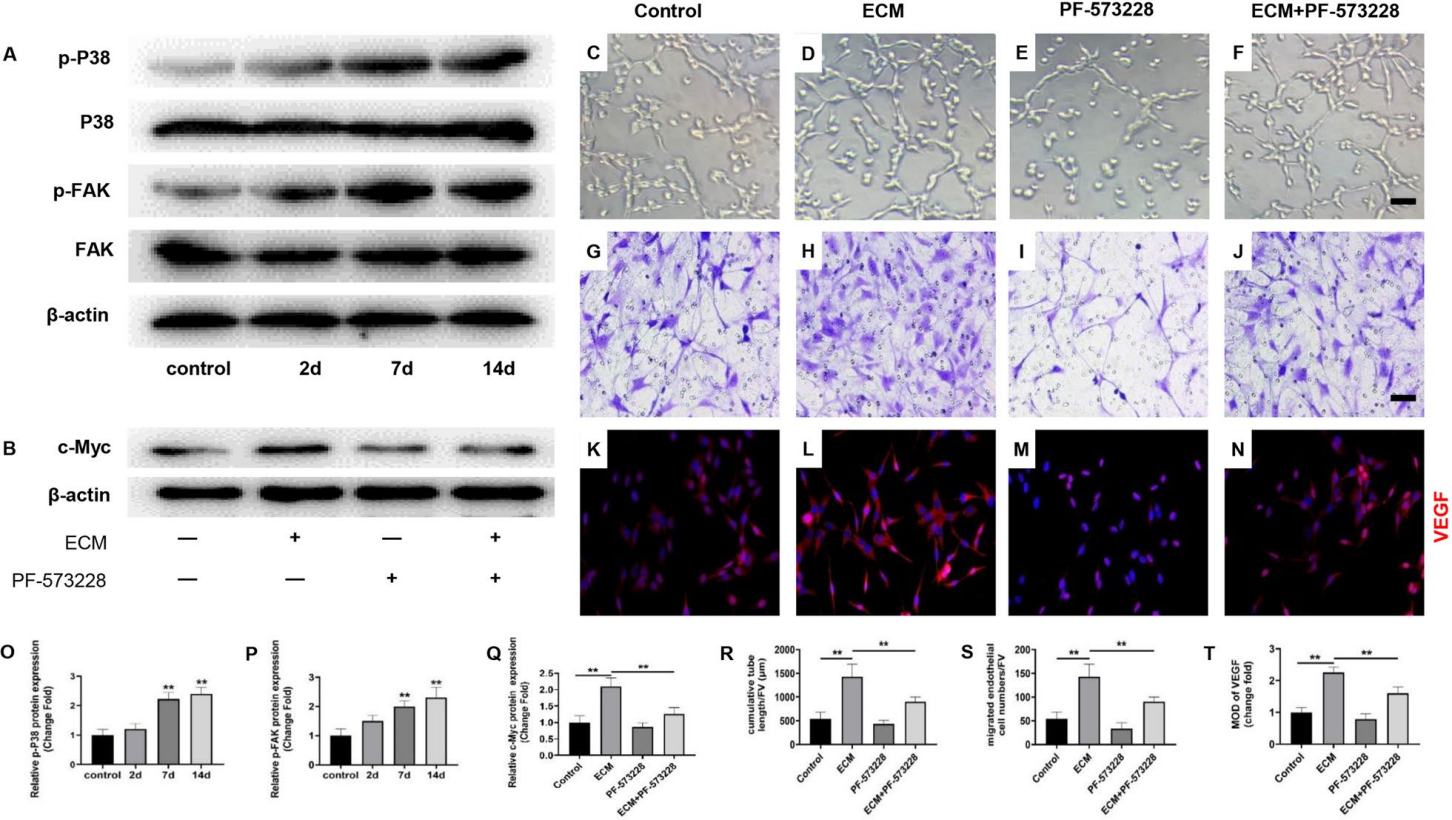
Focal adhesion kinase (FAK) /P38 signaling pathway mediated the effect of WJ-MSCs ECM on HUVECs. **a, b, o–q** Protein expression of FAK, p-FAK, P38, p-P38, c-Myc in HUVECs was determined by western blotting. Mean ± S.E.M. *n* = 3. **c–f, r** Matrigel tube formation assay images of cumulative tube length. Bar = 50 μm. Mean ± S.E.M., *n* = 3. **g–j, s** Transwell for HUVECs migration assay and quantitative analysis of migrated HUVECs numbers. Bar = 50 μm. Mean ± S.E.M., *n* = 3. **k–n, t** Immunofluorescently stained for vascular endothelial growth factor (VEGF) of HUVECs (magnification: × 200). Bar = 50 μm. Mean ± S.E.M., *n* = 3. PF-573228: FAK inhibitors. The *P* value was calculated using one-way ANOVA and independent samples *t*-test. *P < 0.05, **P < 0.01 versus control

The activation of FAK/P38 signaling pathway mediates the synthesis of several key genes during angiogenesis, which is an indispensable process in angiogenesis [27]. Moreover, it has been shown that the activation of FAK/P38 signaling pathway could promote the expression of c-Myc [28]. Therefore, to further explore the mechanism for WJ-MSCs ECM regulating the expression of c-Myc, we detected the expression of FAK/P38 signaling pathway. The results showed that WJ-MSCs ECM significantly promoted the protein expression of p-FAK and p-P38 (Fig. 4a, o–p, *P* < 0.01). Next, we the co-cultured HUVECs with WJ-MSCs ECM after pretreating with FAK/P38 signaling pathway inhibitor PF-573228. The results showed FAK inhibitors significantly reversed the WJ-MSCs ECM-induced increase of HUVECs tube formation and migration (Fig. 4c–f, g–j, r, s, *P* < 0.01) and VEGF expression (Fig. 4k–n, t, *P* < 0.01). Meanwhile, FAK inhibitors PF-573228 significantly reversed the WJ-MSCs ECM-induced increase expression of c-Myc (Fig. 4b, q, *P* < 0.01). These results suggested that WJ-MSCs ECM promoted the expression of c-Myc via FAK/P38 signaling pathway.

**Fig. 5:**
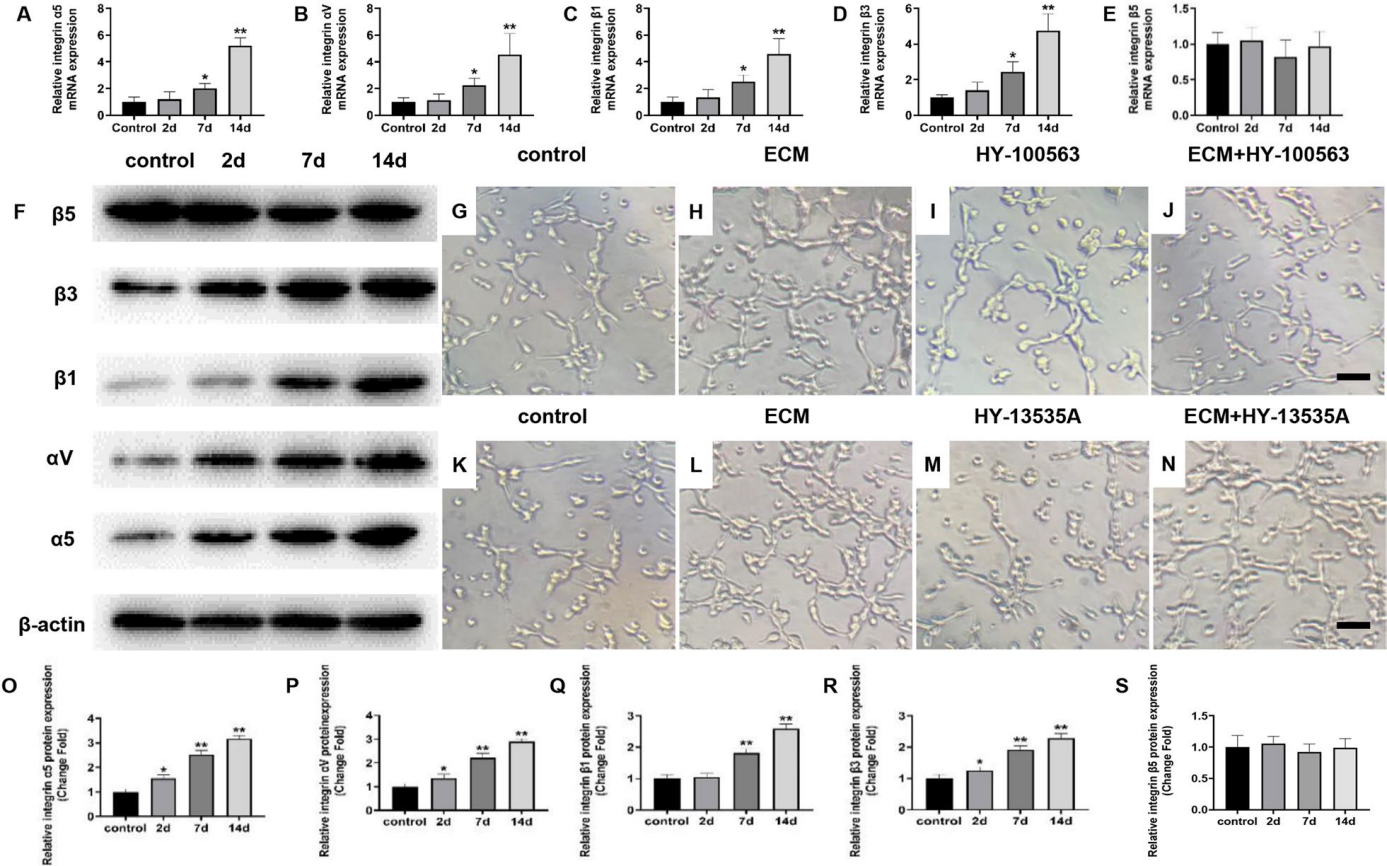
Integrin αVβ3 mediated the effect of WJ-MSCs ECM on HUVECs. **a–e** mRNA expressions of integrin α5, αV, β1, β3, and β5 in HUVECs were determined by RT-qPCR. Mean ± S.E.M.,* n* = 5. **f, o–s** Protein expressions of FAK, p-FAK, P38, p-P38 and VEGF in HUVECs were determined by western blotting. Mean ± S.E.M. *n* = 3. **g**–**n** Matrigel tube formation assay images of cumulative tube length. Bar = 50 μm. HY-100563: integrin αVβ3 specific antagonist. HY-13535A: integrin α5β1 specific antagonist. The *P* value was calculated using one-way ANOVA and independent samples *t*-test. *P < 0.05, **P < 0.01 versus control

Integrins, the transmembrane proteins, are the main receptors for ECM to mediate endothelial cell proliferation and migration [29]. Therefore, to further explore the molecular mechanism of WJ-MSCs ECM regulating FAK/P38 signaling pathway, we detected the expression of integrins in HUVECs cultured with ECM. The results showed that WJ-MSCs ECM promoted the mRNA and protein expression of integrin αVβ3 and integrin α5β1 in a time-dependent manner, but did not affect the expression of integrin α5β5 (Fig. 5a–f, o–s, *P* < 0.05 or *P* < 0.01). These results suggested that WJ-MSCs ECM might regulate intracellular function of HUVECs mainly via integrin αVβ3 and integrin α5β1. Therefore, to further determine the function of integrin αVβ3 and integrin α5β1, HUVECs were pretreated with integrin αVβ3 antagonist HY-100563 and integrin α5β1 antagonist HY-13535A, respectively. Then, the pretreated HUVECs were co-cultured with WJ-MSCs ECM. The results showed that compared with ECM group, ECM + HY100563 group significantly inhibited the tube formation ability of HUVECs (Fig. 5g–j). However, compared with ECM group, ECM + HY13535A did not change the tube formation ability of HUVECs (Fig. 5k–n). These results suggested that WJ-MSCs ECM promoted HUVECs angiogenesis mainly through integrin αVβ3.

**Fig. 6:**
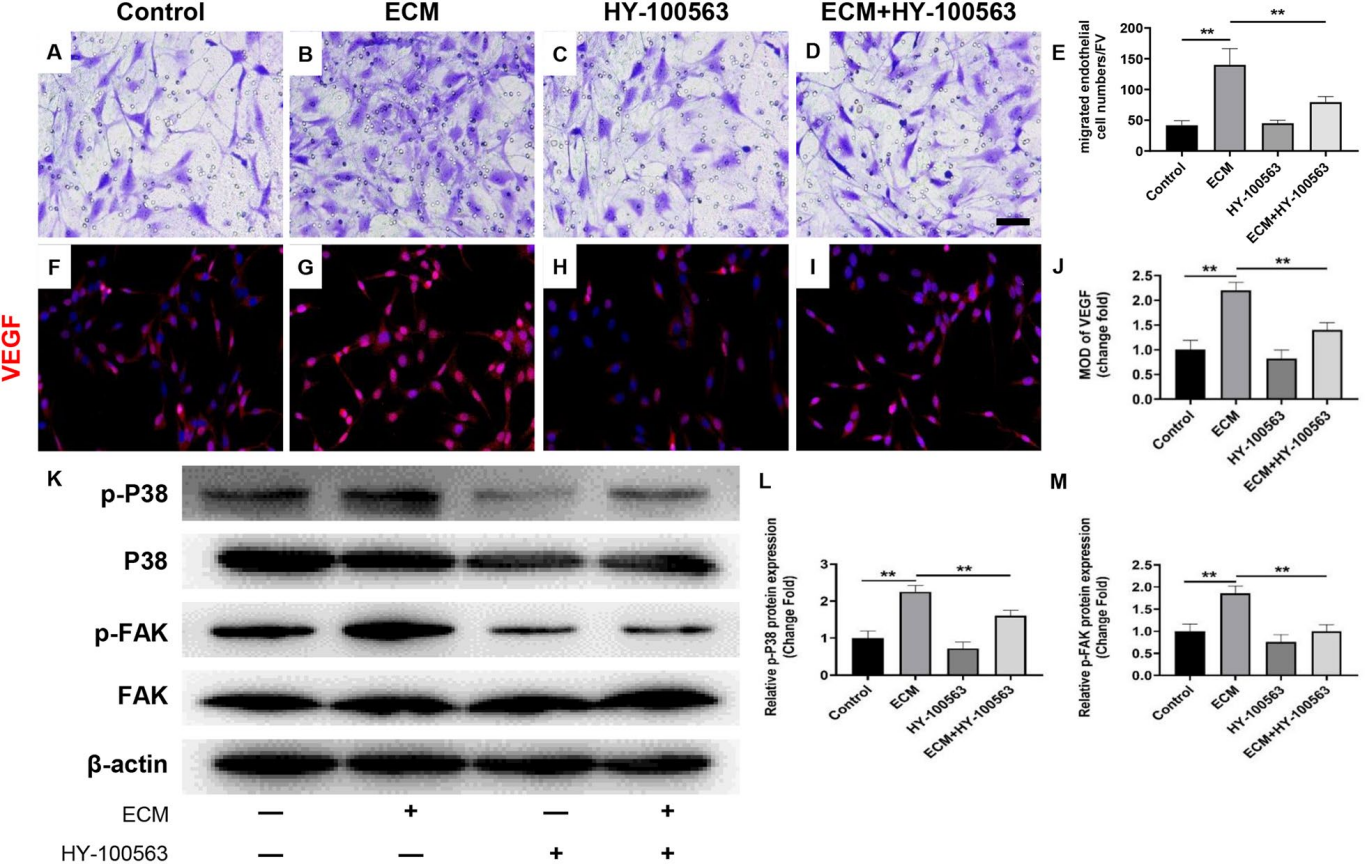
Integrin αVβ3 mediated the activation of FAK/P38 signaling pathway induced by WJ-MSCs ECM in HUVECs. **a–e** Transwell for HUVECs migration assay and quantitative analysis of migrated HUVECs numbers. Bar = 50 μm. Mean ± S.E.M., *n* = 3. **f–j** Immunofluorescently stained for VEGF of HUVECs (magnification: × 200). Mean ± S.E.M., *n* = 3. HY-100563: Integrin αVβ3 specific antagonist. **k–m** Protein expressions of FAK, p-FAK, P38 and p-P38 in HUVECs were determined by western blotting. Mean ± S.E.M. *n* = 3. The *P* value was calculated using one-way ANOVA and independent samples *t*-test. *P < 0.05, **P < 0.01 versus control

Then, we detected the migration function and VEGF expression after antagonizing integrin αVβ3. The results showed that antagonizing integrin αVβ3 significantly reversed the WJ-MSCs ECM-induced increased migration of HUVECs (Fig. 6a–e, *P* < 0.01). Besides, antagonizing integrin αVβ3 significantly reversed the WJ-MSCs ECM-induced increased expression of VEGF (Fig. 6f–j, *P* < 0.01). Also, antagonizing integrin αVβ3 significantly reversed the WJ-MSCs ECM-induced increased expression of p-FAK and p-P38 (Fig. 6k–m, *P* < 0.01). These results suggested that integrin αVβ3 mediated the activation of FAK/P38 signaling pathway in HUVECs induced by WJ-MSCs ECM.

## Discussion

Angiogenesis refers to the generation of new capillaries from the original capillary network, which plays an important role in embryo development and wound healing [30]. However, insufficient angiogenesis is widespread in osteopathic diseases such as osteoporosis, fracture, necrosis of femoral head and tendon injury [31–34]. Due to the insufficient angiogenesis, the lack of oxygen and nutrients in the injured area leads to the obstruction of cell proliferation and matrix synthesis, which ultimately leads to delayed healing or even non-healing [35]. Therefore, it is of great significance to explore the therapeutic means and mechanism of promoting angiogenesis. The ECM is an acellular three-dimensional macromolecular network composed of collagen, fibronectin, laminin and several other glycoproteins. Recent studies suggest that ECM could provide an environment for cell survival, and guide the behavior of cell renewal and self-differentiation [36]. In addition, studies have found that ECM could interact with surrounding cells and regulate cell functions, thus promoting cell proliferation [37]. Therefore, ECM derived from MSCs may be a good mediator for promoting angiogenesis. WJ-MSCs are immersed in ground substance that is rich in collagen, and also containing numerous sulfated glycosaminoglycans. ECMs derived from WJ-MSCs retain the functional properties of their native environment and store signaling molecules [38]. *Letizia *et al*.* found that the ECM derived from WJ-MSC could improve the behavior of cells from degenerated intervertebral disc [36]. Therefore, the ECM derived from WJ-MSCs is becoming increasingly proposed as an ideal medium for promoting tissue regeneration.

In this study, ECM derived from WJ-MSCs was obtained and identified as previously described [19]. The identification results showed that the nuclei of WJ-MSCs disappeared after decellularization. In addition, the cell membrane structure of WJ-MSCs broke, and the main component of ECM, COL I, escaped from the cells and distributed evenly outside the cells. Also, we found the ECM derived from WJ-MSCs expressed a large number of Fibronectin and Laminin, which was similar to those reported in previous study [39]. Further, we investigated the angiogenesis promoting function of WJ-MSCs ECM in vitro. The results showed that WJ-MSCs ECM promoted the angiogenesis of HUVECs in a time-dependent manner. Therefore, the ECM derived from WJ-MSCs could effectively promote the angiogenesis of HUVECs in vitro.

Angiogenesis is mainly composed of endothelial cell proliferation, migration and tube formation [3]. VEGF mainly regulates the migration of endothelial cells during angiogenesis [3]. The overexpression of VEGF promotes the angiogenesis by acting in a paracrine fashion on vicinal endothelium [40]. Epigenetic regulation has been defined as the changes in gene expression that occur without a change in the DNA sequence [41]. Histone modification is a common type of epigenetic form and includes histone acetylation, which can regulate the expression of target genes [42]. The high levels of histone acetylation facilitate chromatin opening and thus promote gene expression [42]. Also, previous studies have found that epigenetic modifications play important roles in the gene expression and functional changes induced by ECM [43, 44]. In this study, we found that the WJ-MSCs ECM could increase the expression of VEGF in HUVECs accompanied by increased histone acetylation levels in the promoter region. These suggested that the WJ-MSCs ECM might regulate the expression of VEGF via histone modification which triggered angiogenesis in HUVECs.

C‐Myc is often recognized as a crucial transcription regulator and acts as a forceful impulse on the expression of targeted genes [45, 46]. It could regulate the expression of targeted genes via recruit epigenetic enzymes [47]. In addition, the expression knockdown of c-Myc could inhibit angiogenesis [48]. In this study, we found that the WJ-MSCs ECM increased the expression of c-Myc in HUVECs. Furthermore, the c-Myc expression silencing also reversed the WJ-MSCs ECM-induced increase of VEGF mRNA and histone acetylation level in promoter region. Therefore, c-Myc increased the expression of VEGF via histone modification induced by the WJ-MSCs ECM. The histone acetylation is regulated by histone acetylase and histone deacetylases. P300 is a histone acetylation enzyme that can improve the acetylation level of the target gene promoter region [49]. In this study, we found that WJ-MSCs ECM promoted P300 expression in a time-dependent manner. The expression silence of P300 significantly reversed the changes in the expression of VEGF and the histone acetylation levels in the VEGF promoter region induced by WJ-MSCs ECM. Further, we found that c-Myc interacted with P300 through Co-IP assay. Therefore, to sum up, c-Myc improved the acetylation level of VEGF promoter region by recruiting P300, thus promoting the expression of VEGF in HUECs in the condition of co-culturing with WJ-MSCs ECM.

FAK is a key regulator of focal adhesion signaling and the activation of FAK known be related with expression of several angiogenic cytokines [27, 50]. Moreover, it has been proved that the activation of FAK could lead to phosphorylation of P38, and the p-P38 triggered the translation of c-Myc [28]. In this study, we found that WJ-MSCs ECM promoted activation of FAK/P38 signaling pathway in HUVECs in a time-dependent manner. Furthermore, the inhibition of FAK/P38 signaling pathway reversed the increased-expression of c-Myc induced by WJ-MSCs ECM. These suggested that WJ-MSCs ECM promoted the expression activation of FAK/P38 signaling pathway. Integrin is receptor that mediate recognition and adhesion between cells and between cells and extracellular matrix [51]. Integrin delivers signals to cells by binding to protein components of the extracellular matrix such as collagen, fibronectin and osteopontin [52]. In this study, we found that WJ-MSCs ECM promoted integrin αVβ3, integrin α5β1 expression in a time-dependent manner. Further, we antagonized integrin αVβ3 and integrin α5β1 by specific receptor antagonists, respectively. The results showed that integrin αVβ3 antagonist significantly reversed the changes in HUVECs angiogenesis ability induced by WJ-MSCs ECM, while integrin α5β1 antagonist did not reverse the changes in HUVECs angiogenesis ability induced by WJ-MSCs ECM. Not only that, that integrin αVβ3 antagonist significantly reversed the activation of FAK/P38 signaling pathway in HUVECs. Thus, we speculated that integrin αVβ3 mediated the activation of FAK/P38 signaling pathway and the WJ-MSCs ECM-induced angiogenesis increasing in HUVECs.

## Conclusion

**Fig. 7:**
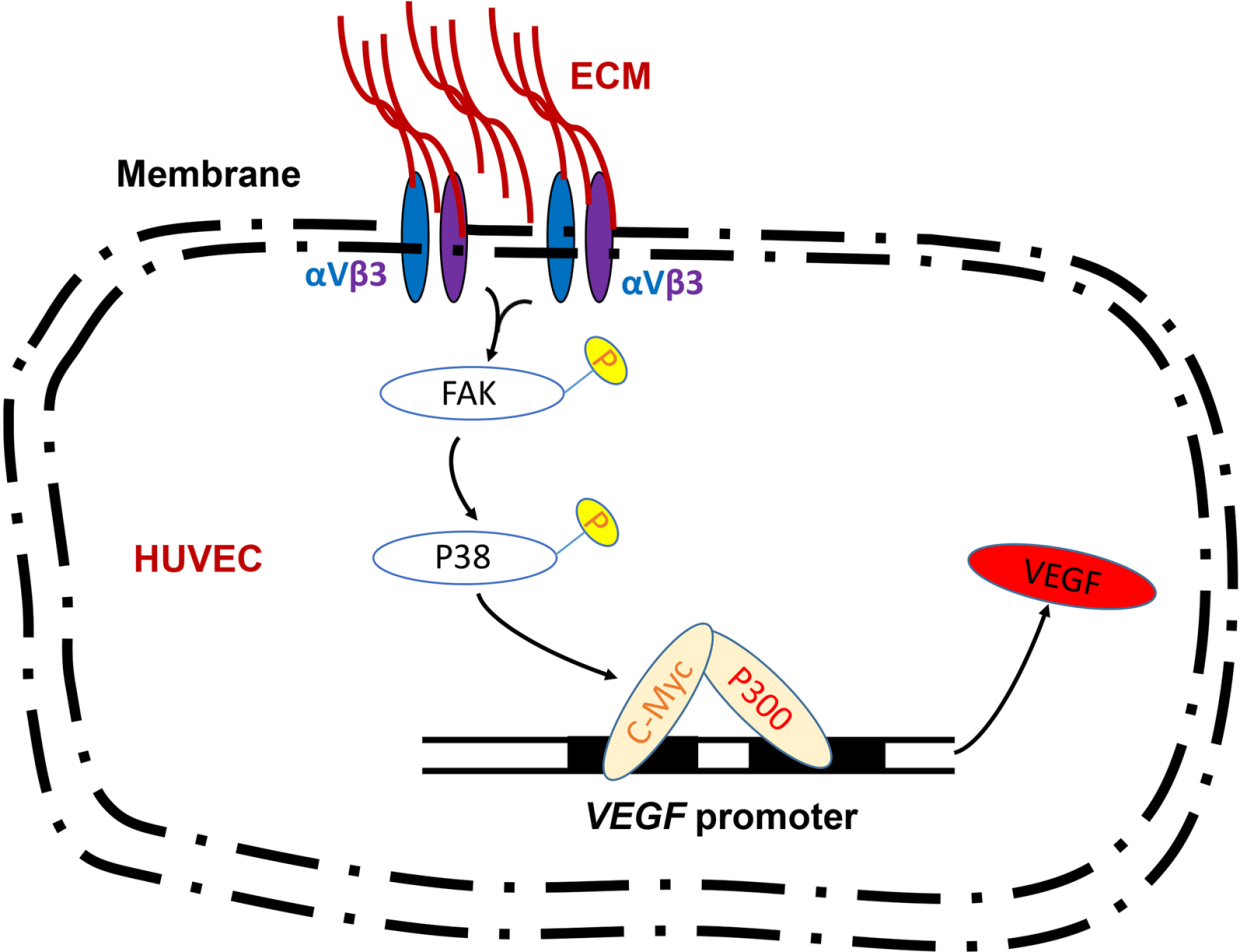
ECM derived from WJ-MSCs promoted angiogenesis via integrin αVβ3/c-Myc/P300/VEGF. ECM: Extracellular matrix. αVβ3: integrin αVβ3. HUVEC: human umbilical vein endothelial cell. c-Myc: cellular-Myc. VEGF: vascular endothelial growth factor

In this study, we found for the first time that WJ-MSCs ECM could improve the angiogenesis ability of HUVECs with a time-dependent manner in vitro. Mechanically, WJ-MSCs ECM activated the FAK/P38 signaling pathway via integrin αVβ3, which further promoted the expression of the c-Myc. Further, c-Myc increased the histone acetylation levels of VEGF promoter by recruiting P300, which ultimately promoting VEGF expression (Fig. 7). Moreover, due to the persistence of epigenetic imprinting, the WJ-MSCs ECM-pretreated endothelial cells might be a new therapeutic approach to promote angiogenesis during tissue repair and bone regeneration.

## Data Availability

The data that support the findings of this study are available from the corresponding authors upon reasonable request.

## Abbreviations

MxxSCs: Mesenchymal stem cells
MxxSCs-ECM: MxxSCs-derived extracellular matrix
WJ-MSCs: Wharton's Jelly-derived mesenchymal stem cells
HUVECs: Human umbilical vein endothelial cells
FAK: Focal adhesion kinase
VEGF: Vascular endothelial growth factor
c-Myc: Cellular (c)-Myc
p-FAK: Phospho-FAK
p-P38: Phospho-P38
COL I: Collagen I
ChIP: Chromatin Immunoprecipitation
Co-IP: Co-Immunoprecipitation
H3K9ac: Histone 3 lysine 9 acetylation

## References

[1] Huang S, Lei D, Yang Q, Yang Y, Jiang C, Shi H, Qian B, Long Q, Chen W, Chen Y, Zhu L, Yang W, Wang L, Hai W, Zhao Q, You Z, Ye X (2021). A perfusable, multifunctional epicardial device improves cardiac function and tissue repair. Nat Med.

[2] Park H, Yamamoto H, Mohn L, Ambühl L, Kanai K, Schmidt I, Kim KP, Fraccaroli A, Feil S, Junge HJ, Montanez E, Berger W, Adams RH (2019). Integrin-linked kinase controls retinal angiogenesis and is linked to Wnt signaling and exudative vitreoretinopathy. Nat Commun.

[3] Lamalice L, Boeuf FL, Huot JJCR (2007). Endothelial cell migration during. Angiogenesis.

[4] Xie H, Cui Z, Wang L, Xia Z, Hu Y, Xian L, Li C, Xie L, Crane J, Wan M, Zhen G, Bian Q, Yu B, Chang W, Qiu T, Pickarski M, Duong LT, Windle JJ, Luo X, Liao E, Cao X (2014). PDGF-BB secreted by preosteoclasts induces angiogenesis during coupling with osteogenesis. Nat Med.

[5] Percival CJ, Richtsmeier JT (2013). Angiogenesis and intramembranous osteogenesis. Dev Dyn.

[6] Hughes S. The role of the blood supply in tissue reconstruction. 2012.

[7] Beldi G, Bahiraii S, Lezin C, Nouri Barkestani M, Abdelgawad ME, Uzan G, Naserian S (2020). TNFR2 is a crucial hub controlling mesenchymal stem cell biological and functional properties. Front Cell Dev Biol.

[8] Sart S, Jeske R, Chen X, Ma T, Li YJTEPBR. Engineering stem cell-derived extracellular matrices: decellularization and biological function. 2020; 26. 10.1089/ten.TEB.2019.0349 32220216

[9] Kaukonen R, Jacquemet G, Hamidi H, Ivaska J (2017). Cell-derived matrices for studying cell proliferation and directional migration in a complex 3D microenvironment. Nat Protoc.

[10] Sart S, Ma T, Li Y (2014). Extracellular matrices decellularized from embryonic stem cells maintained their structure and signaling specificity. Tissue Eng A.

[11] Sart S, Yan Y, Li Y, Lochner E, Zeng C, Ma T, Li Y (2016). Crosslinking of extracellular matrix scaffolds derived from pluripotent stem cell aggregates modulates neural differentiation. Acta Biomater.

[12] Lech W, Sarnowska A, Kuczynska Z, Dabrowski F, Figiel-Dabrowska A, Domanska-Janik K, Buzanska L, Zychowicz M (2020). Biomimetic microenvironmental preconditioning enhance neuroprotective properties of human mesenchymal stem cells derived from Wharton's Jelly (WJ-MSCs). Sci Rep.

[13] Jadalannagari S, Converse G, McFall C, Buse E, Filla M, Villar MT, Artigues A, Mellot AJ, Wang J, Detamore MS, Hopkins RA, Aljitawi OS (2017). Decellularized Wharton's Jelly from human umbilical cord as a novel 3D scaffolding material for tissue engineering applications. PLoS ONE.

[14] Kehtari M, Beiki B, Zeynali B, Hosseini FS, Soleimanifar F, Kaabi M, Soleimani M, Enderami SE, Kabiri M, Mahboudi H (2019). Decellularized Wharton's jelly extracellular matrix as a promising scaffold for promoting hepatic differentiation of human induced pluripotent stem cells. J Cell Biochem.

[15] Penolazzi L, Pozzobon M, Bergamin LS, D’Agostino S, Francescato R, Bonaccorsi G, De Bonis P, Cavallo M, Lambertini E, Piva R. Extracellular matrix from decellularized Wharton’s Jelly improves the behavior of cells from degenerated intervertebral disc. 2020; 8. 10.3389/fbioe.2020.00262 PMC7118204 32292779

[16] Wu CH, Ko JL, Pan HH, Chiu LY, Kang YT, Hsiao YP (2019). Ni-induced TGF-β signaling promotes VEGF-a secretion via integrin β3 upregulation. J Cell Physiol.

[17] Liu G, Wu H, Chen L, Xu J, Wang M, Li D, Lu P (2017). Effects of interleukin-17 on human retinal vascular endothelial cell capillary tube formation in vitro. Mol Med Rep.

[18] Ahn J, Park EM, Kim BJ, Kim JS, Choi B, Lee SH, Han I (2015). Transplantation of human Wharton's jelly-derived mesenchymal stem cells highly expressing TGFβ receptors in a rabbit model of disc degeneration. Stem Cell Res Ther.

[19] Li J, Pei M (2018). A protocol to prepare decellularized stem cell matrix for rejuvenation of cell expansion and cartilage regeneration. Methods Mol Biol (Clifton, NJ).

[20] Risau, Nature WJ. Mechanisms of angiogenesis. 1997. 10.1038/386671a0 9109485

[21] Carmeliet P, Jain RKJN. Molecular mechanisms and clinical applications of angiogenesis. 2011; 473: 298–307. 10.1038/nature10144 PMC4049445 21593862

[22] Huang X, Sun J, Chen G, Niu C, Wang Y, Zhao C, Sun J, Huang H, Huang S, Liang Y, Shen Y, Cong W, Jin L, Zhu Z (2019). Resveratrol promotes diabetic wound healing via SIRT1-FOXO1-c-Myc signaling pathway-mediated angiogenesis. Front Pharmacol.

[23] Ma J, Tang W, Gu R, Hu F, Zhang L, Wu J, Xu G (2020). SHP-2-induced activation of c-Myc is involved in PDGF-B-regulated cell proliferation and angiogenesis in RMECs. Front Physiol.

[24] Hu L, Lv X, Li D, Zhang W, Ran G, Li Q, Hu J (2021). The anti-angiogenesis role of FBXW7 in diabetic retinopathy by facilitating the ubiquitination degradation of c-Myc to orchestrate the HDAC2. J Cell Mol Med.

[25] Deans C, Maggert KA (2015). What do you mean, "epigenetic"?. Genetics.

[26] Narita T, Weinert BT, Choudhary C (2019). Functions and mechanisms of non-histone protein acetylation. Nat Rev Mol Cell Biol.

[27] Lugano R, Vemuri K, Yu D, Bergqvist M, Smits A, Essand M, Johansson S, Dejana E, Dimberg A (2018). CD93 promotes β1 integrin activation and fibronectin fibrillogenesis during tumor angiogenesis. J Clin Investig.

[28] Duperret EK, Dahal A, Ridky TW (2015). Focal-adhesion-independent integrin-αv regulation of FAK and c-Myc is necessary for 3D skin formation and tumor invasion. J Cell Sci.

[29] Creamer D, Sullivan D, Bicknell R, Barker JJA. Angiogenesis in psoriasis. 2002; 5: 231–6. 10.1023/a:1024515517623 12906316

[30] Jetten N, Verbruggen S, Gijbels MJ, Post MJ, De Winther MP, Donners MM (2014). Anti-inflammatory M2, but not pro-inflammatory M1 macrophages promote angiogenesis in vivo. Angiogenesis.

[31] Yang W, Zhu W, Yang Y, Guo M, Qian H, Jiang W, Chen Y, Lian C, Xu Z, Bai H, Chen T, Zhang J (2021). Exosomal miR-100-5p inhibits osteogenesis of hBMSCs and angiogenesis of HUVECs by suppressing the BMPR2/Smad1/5/9 signalling pathway. Stem Cell Res Ther.

[32] Zaidi M, Lizneva D, Yuen T (2021). The role of PDGF-BB in the bone-vascular relationship during aging. J Clin Investig.

[33] Dadwal UC, Bhatti FUR, Awosanya OD, Nagaraj RU, Perugini AJ, Sun S, Valuch CR, de Andrade SC, Mendenhall SK, Tewari NP, Mostardo SL, Nazzal MK, Battina HL, Zhou D, Kanagasabapathy D, Blosser RJ, Mulcrone PL, Li J, Kacena MA (2021). The effects of bone morphogenetic protein 2 and thrombopoietin treatment on angiogenic properties of endothelial cells derived from the lung and bone marrow of young and aged, male and female mice. FASEB J.

[34] Moreno SE, Massee M, Koob TJ. Dehydrated human amniotic membrane regulates tenocyte expression and angiogenesis in vitro: implications for a therapeutic treatment of tendinopathy. J Biomed Mater Res B Appl Biomater. 2021. 10.1002/jbm.b.34951 PMC9292862 34611976

[35] Bikfalvi A (2006). Angiogenesis: health and disease. Ann Oncol.

[36] Oliveira H, Médina C, Labrunie G, Dusserre N, Catros S, Magnan L, Handschin C, Stachowicz ML, Fricain JC, L'Heureux N. Cell-assembled extracellular matrix (CAM): a human biopaper for the biofabrication of pre-vascularized tissues able to connect to the host circulationin vivo. Biofabrication. 2021;14. 10.1088/1758-5090/ac2f81 34695012

[37] Wang Z, Han L, Sun T, Ma J, Sun S, Ma L, Wu B (2020). Extracellular matrix derived from allogenic decellularized bone marrow mesenchymal stem cell sheets for the reconstruction of osteochondral defects in rabbits. Acta Biomater.

[38] Sart S, Jeske R, Chen X, Ma T, Li Y (2020). Engineering stem cell-derived extracellular matrices: decellularization, characterization, and biological function. Tissue Eng Part B Rev.

[39] Correction: Decellularized Wharton's Jelly from human umbilical cord as a novel 3D scaffolding material for tissue engineering applications. PLoS ONE. 2017;12: e0173827. 10.1371/journal.pone.0172098 PMC5319682 28222169

[40] Chung J, Bachelder RE, Lipscomb EA, Shaw LM, Mercurio AM (2002). Integrin (alpha 6 beta 4) regulation of eIF-4E activity and VEGF translation: a survival mechanism for carcinoma cells. J Cell Biol.

[41] Ling C, Rnn TJCM. Epigenetics in human obesity and type 2 diabetes. 2019; 29. 10.1016/j.cmet.2019.03.009 PMC6509280 30982733

[42] Shafabakhsh R, Aghadavod E, Ghayour‐Mobarhan M, Ferns G, Asemi ZJJoCP. Role of histone modification and DNA methylation in signaling pathways involved in diabetic retinopathy. 2019; 234. 10.1002/jcp.27844 30515789

[43] Che LH, Liu JW, Huo JP, Luo R, Xu RM, He C, Li YQ, Zhou AJ, Huang P, Chen YY, Ni W, Zhou YX, Liu YY, Li HY, Zhou R, Mo H, Li JM (2021). A single-cell atlas of liver metastases of colorectal cancer reveals reprogramming of the tumor microenvironment in response to preoperative chemotherapy. Cell Discov.

[44] Aloia L. The influence of tissue spatial geometry and functional organisation on liver regeneration. Semin Cell Dev Biol. 2021. 10.1016/j.semcdb.2021.09.011 34563460

[45] Wierstra I, Alves J (2008). The c-myc promoter: still MysterY and challenge. Adv Cancer Res.

[46] Gabay M, Li Y, Felsher DW. MYC activation is a hallmark of cancer initiation and maintenance. Cold Spring Harbor Perspect Med. 2014; 4. 10.1101/cshperspect.a014241 PMC4031954 24890832

[47] Zhao J, Lin H, Huang K. Mesenchymal stem cell-derived extracellular vesicles transmitting MicroRNA-34a-5p suppress tumorigenesis of colorectal cancer through c-MYC/DNMT3a/PTEN axis. Mol Neurobiol. 2021. 10.1007/s12035-021-02431-9 PMC8786758 34623601

[48] Debnath S, Mukherjee A, Saha D, Dash J, Chatterjee TK (2021). Poly-l-Lysine inhibits VEGF and c-Myc mediated tumor-angiogenesis and induces apoptosis in 2D and 3D tumor microenvironment of both MDA-MB-231 and B16F10 induced mice model. Int J Biol Macromol.

[49] Jiang S, Ren J, Xu Q, Zou X, Li Y, Zhang CY. Simultaneous single-molecule detection of the acetyltransferase and crotonyltransferase activities of histone acetylation writer p300. Chem Commun (Cambridge, England). 2021. 10.1039/d1cc05449j 34693944

[50] Wu S, Chen M, Huang J, Zhang F, Lv Z, Jia Y, Cui YZ, Sun LZ, Wang Y, Tang Y, Verhoeft KR, Li Y, Qin Y, Lin X, Guan XY, Lam KO (2021). ORAI2 promotes gastric cancer tumorigenicity and metastasis through PI3K/Akt signaling and MAPK-dependent focal adhesion disassembly. Can Res.

[51] Sedlář A, Trávníčková M, Bojarová P, Vlachová M, Slámová K, Křen V, Bačáková L (2021). Interaction between Galectin-3 and integrins mediates cell-matrix adhesion in endothelial cells and mesenchymal stem cells. Int J Mol Sci.

[52] Goes LR, Sajani A, Sivro A, Olowojesiku R, Ray JC, Perrone I, Yolitz J, Girard A, Leyre L, Wibmer CK, Morris L, Gorini G, Franchini G, Mason RD, Roederer M, Mehandru S, Soares MA, Cicala C, Fauci AS, Arthos J (2020). The V2 loop of HIV gp120 delivers costimulatory signals to CD4(+) T cells through Integrin α(4)β(7) and promotes cellular activation and infection. Proc Natl Acad Sci USA.

